# Relationship between household air pollution and lung cancer in never smokers in high-income countries: a systematic review

**DOI:** 10.1136/bmjopen-2024-093870

**Published:** 2025-06-20

**Authors:** Bría Joyce McAllister, Rushad Malhotra Mukhtyar, Samuel Cai, Karen Brown, Selina Lock, Sam Khan

**Affiliations:** 1 University of Leicester College of Life Sciences, Leicester, UK; 2 Leicester Cancer Research Centre, University of Leicester, Leicester, UK; 3 University of Leicester, Leicester, UK; 4 University Hospitals of Leicester NHS Trust, Leicester, UK; 5 Department of Health Sciences, University of Leicester, Leicester, UK

**Keywords:** Lung Diseases, EPIDEMIOLOGY, Respiratory tract tumours, PUBLIC HEALTH, Systematic Review, Epidemiology

## Abstract

**Abstract:**

**Objectives:**

Lung cancer is increasingly being diagnosed in non-smokers, with mounting evidence that household air pollution is a potential factor. Environmental risk factors for lung cancer in never-smokers (LCINS) in relation to combustion of biomass for heating and cooking in low-middle-income countries (LMICs) have been extensively explored. However, such evidence in high-income countries (HICs) is limited. We conducted a systematic review to explore potential relationships between exposure to cooking fumes, a type of household air pollution, and lung cancer, specifically in relation to never-smokers in HICs.

**Design:**

Systematic review and narrative synthesis using the Critical Analysis Skills Programme (CASP) guidelines for case–control studies.

**Data sources:**

Embase, Scopus, the Cochrane library and CINAHL were searched, from inception to March 2024. Reference lists of articles were hand searched for additional papers.

**Eligibility criteria:**

Case–control studies focusing on household air pollution and its impact on LCINS in HICs were included.

**Data extraction and synthesis:**

Two independent reviewers searched, screened and coded included studies using a bespoke table. Quality of evidence was assessed in the selected studies using the CASP tool for case–control studies. Retained studies used different exposure assessment and reporting methods which were sufficiently heterogeneous to preclude meta-analysis; therefore, narrative synthesis was performed.

**Results:**

Three papers were included, with a total of 3734 participants. All studies were conducted in Taiwan or Hong Kong, focusing on Chinese women using traditional Chinese cooking methods. All three found a dose/response correlation between exposure to cooking fumes and the risk of developing LCINS.

Chen *et al* assessed the risk of lung cancer risk by ‘cooking time-years’, measuring exposure to cooking fumes over a participant’s lifetime, citing OR 3.17 (95% CI 1.34 to 7.68) for the highest levels of exposure. Yu *et al* used ‘cooking dish-years’ as a measure of exposure to cooking fumes, with OR 8.09 (95% CI 2.57 to 25.45) for the highest exposure levels, while Ko *et al* found that the number of dishes cooked daily was a greater indicator of risk than the number of cooking years, citing a threefold increased risk of lung cancer among women who cooked three meals per day compared with those who cooked one (OR 3.1, 95% CI 1.6 to 6.2).

Ventilation hoods were found to have a protective effect against LCINS with adjusted ORs of 0.49 (95% CI 0.32 to 0.76).

**Conclusions:**

This review of three studies found a possible association between exposure to cooking fumes and the risk of developing LCINS in high-income settings. This corroborates the substantial body of evidence that links cooking fume exposure to LCINS in LMICs, with definitive confirmation of the exposure hazards.

**PROSPERO registration number:**

CRD42024524445.

**STRENGTHS AND LIMITATIONS OF THIS STUDY**

- A specialist librarian was consulted during the development of the search strings.
- All papers were independently screened by two authors.
- Data extraction was completed by the first author and double-checked by the second author, with the quality of evidence assessed using the Critical Analysis Skills Programme tool. The study was registered with International Prospective Register of Systematic Reviews prior to commencement.
- Studies published in non-English language journals may not have been identified by search strings.

## Introduction

 People spend the majority of their time (80%–90%) indoors, whether in their own homes, or in indoor public spaces such as schools, workplaces and public transport, with some vulnerable groups spending significantly longer indoors.^1 2^ Indoor air encompasses a more diverse range of pollutants, and often at higher concentrations, than outdoor air.^3^ Recent initiatives to improve outdoor air quality have highlighted the negative impact of indoor air pollution on health.^1 3 4^ Indoor air quality is influenced by a wide variety of factors including indoor and outdoor sources, personal behaviour, building design, household socioeconomic status and ventilation. Despite this, indoor air has not been researched to the degree that outdoor air has been, with fewer resources dedicated to our understanding of this issue.^2^ The UK Chief Medical Officers report into air pollution (2023) calls for a greater understanding of indoor air pollution coupled with a robust plan for reducing exposure to poor indoor air quality.^2^

Indoor air pollution is a complex issue, with specific factors that apply uniquely to it and differ from outdoor air pollution.^3^ Indoor air shares some pollutants with outdoor air,^5^ for example, particulate matter (PM), and some pollutants may enter from outdoors, becoming trapped indoors.^3^ Research has shown that anywhere between 10% and almost 100% of indoor air pollutants come from outdoors via infiltration.^6^ Exposure to PM in air has been shown to drive lung mutations in human and animal models, leading to increased risk of lung cancer in never-smokers (LCINS).^7^

However, indoor air also contains distinct pollutants which derive directly from household activities such as cooking, heating, cleaning and sweeping, or volatile organic compounds (VOCs) from household furnishings.^3^,^5^ VOCs which are generated by cooking oils have been shown to be mutagenic, containing polycyclic aromatic hydrocarbons (PACs), aldehydes, carbonyl compounds and other mutagens.^7^,^9^

**Figure 1:**
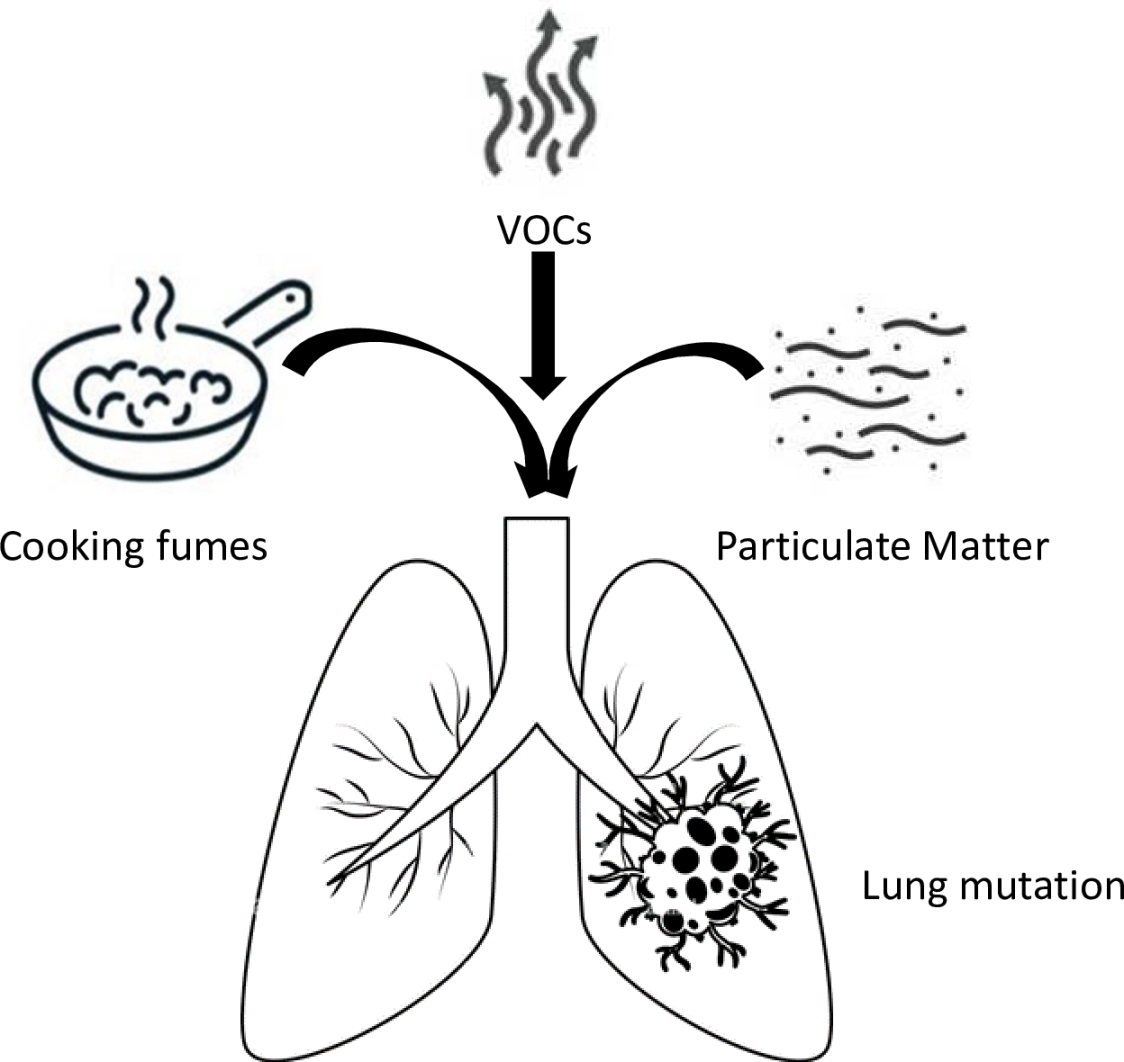
Impact of cooking-related air pollution on lung cancer in never-smokers. VOCs, volatile organic compounds.

The WHO cites household air pollution as being responsible for an estimated 3.2 million deaths in the year 2020, with 237 000 of those deaths being in children under the age of 5 years, who are more likely to spend time in the home and be exposed to household pollution.^10^ Similarly, since activities such as cooking and heating account for a significant proportion of indoor air pollution,^11^ women and children are disproportionately affected.^12 13^ It is estimated that over half of the world’s population relies on biomass fuels for the purposes of cooking and/or heating.^14 15^ It must be noted that the vast majority of the literature on household air pollution comes from low-income and middle-income countries (LMICs) where there is significant reliance on biomass fuels for cooking and heating, and therefore, may not be directly applicable to high-income countries (HICs).^8 15 16^ Exposure to cooking fumes is linked to lung cancer due to the levels of carcinogens that can be produced during the heating of oils to high temperatures.^8 16 17^ Never-smoking patients who present with new adenocarcinoma of the lung are more likely to have been exposed to household air pollution including cooking oil fumes (COFs) and fuel used for cooking purposes.^8 18^ Exposure to PM, VOCs and cooking fumes may increase the risk of developing LCINS (figure 1).

Globally, an estimated 10%–25% of lung cancer diagnoses occur in never-smoking patients.^8^ LCINS can be viewed as a separate entity from smoking-related lung cancer, with different demographics, progression and outcomes.^19 20^ When viewed as a specific disease, LCINS accounts for 6000 UK deaths annually, is the 8th most common cancer in the UK and the 7th leading cause of cancer deaths worldwide.^8 17 19^

Never-smoking patients who are diagnosed with lung cancer tend to be female,^8 21^ younger^19 20^ and most commonly suffering from adenocarcinomas.^22^,^24^ Family history also plays a significant role in understanding the risk of LCINS, with a strong association between lung disease, especially asthma, or a history of lung cancer in a first-degree relative (parent or sibling) leading to a significant increase in the likelihood of a never-smoker developing lung cancer.^16 25 26^ Environmental risk factors for LCINS, particularly household air pollution in relation to combustion of biomass for heating and cooking in LMICs, have been extensively explored.^38 15 24 27^,^31^ However, such evidence in HICs is only emerging in recent years.

The aim of this review is to compile all available evidence on the relationship between exposure to cooking fumes, a type of household air pollution, and lung cancer; specifically in relation to never-smokers in HICs.

## Methods

The systematic review protocol was registered with PROSPERO, the International Prospective Register of Systematic Reviews (Identification number CRD42024524445). In accordance with the Preferred Reporting Items for Systematic Reviews and Meta-Analyses (PRISMA) guidelines, Medline, Embase, Scopus and CINAHL databases were electronically searched. The research question was formulated as a priori: ‘What is the relationship between household air pollution and lung cancer, specifically in relation to never-smokers in high income countries?’, and a Population, Exposure, Comparator and Outcome (PECO) statement was created (table 1). No language restrictions were placed on searches, and translating software was used where required for title and abstract screening. The three retained papers were published in English. We used the PRISMA checklist when writing our report.^32^

**Table 1:** Population, Exposure, Comparator and Outcome table of inclusion and exclusion criteria

Components	Inclusion criteria	Exclusion criteria
Participants	Never smokers, defined as adults who have never smoked, or smoked fewer than 100 cigarettes in their lifetime (1).Participants who were residing in high-income countries (2).	Passive smokers
Exposure	Indoor air pollutionWe define ‘indoor air pollution’ as (a) exposure to polluting fuel used for cooking and heating; (b) exposure to gas fuel for cooking; (c) exposure to mould; (d) exposure to incense burning and (e) exposure to actual measured concentrations of indoor air pollutants.	Radon exposureexposure to passive/secondhand smokingexposure to outdoor or ambient air pollutionoccupational exposures
Comparator	Relative risks, at least adjusted for age and sex, for outcomes per unit increment of indoor pollutant concentrations, or risk in those exposed to indoor air pollution compared with those unexposed.	No comparison groups
Outcome	Diagnosis of lung cancer in non-smoking patients	Diagnosis of lung cancer in smoking patients

### Inclusion/exclusion criteria

We used the list of HICs as defined by the World Bank.^33^ Never-smokers are a key population group for this study and are defined by the Centres for Disease Control and Prevention as ‘An adult who has never smoked, or who has smoked less than 100 cigarettes in his or her lifetime’.^34^ The databases were searched from inception to March 2024.

### Identification and selection of studies

Studies that analysed household air pollution as exposures and LCINS as outcomes were searched. The search was conducted in Medline, Embase, Scopus and CINAHL for relevant studies. The keywords used included “lung cancer”, “LCINS”, “non-smokers”, never-smokers”, “household air pollution”, “indoor air pollution” and “exposure”. A specialist librarian was consulted during the development of the search strings. Additional searches were conducted by scanning reference lists of returned studies in order to capture any relevant papers which had not been found through the database searches. Study selection criteria are given in table 1, which generally follows the PECO approach.^35^ The full search criteria can be found in online supplemental appendix 1.

**Figure 2:**
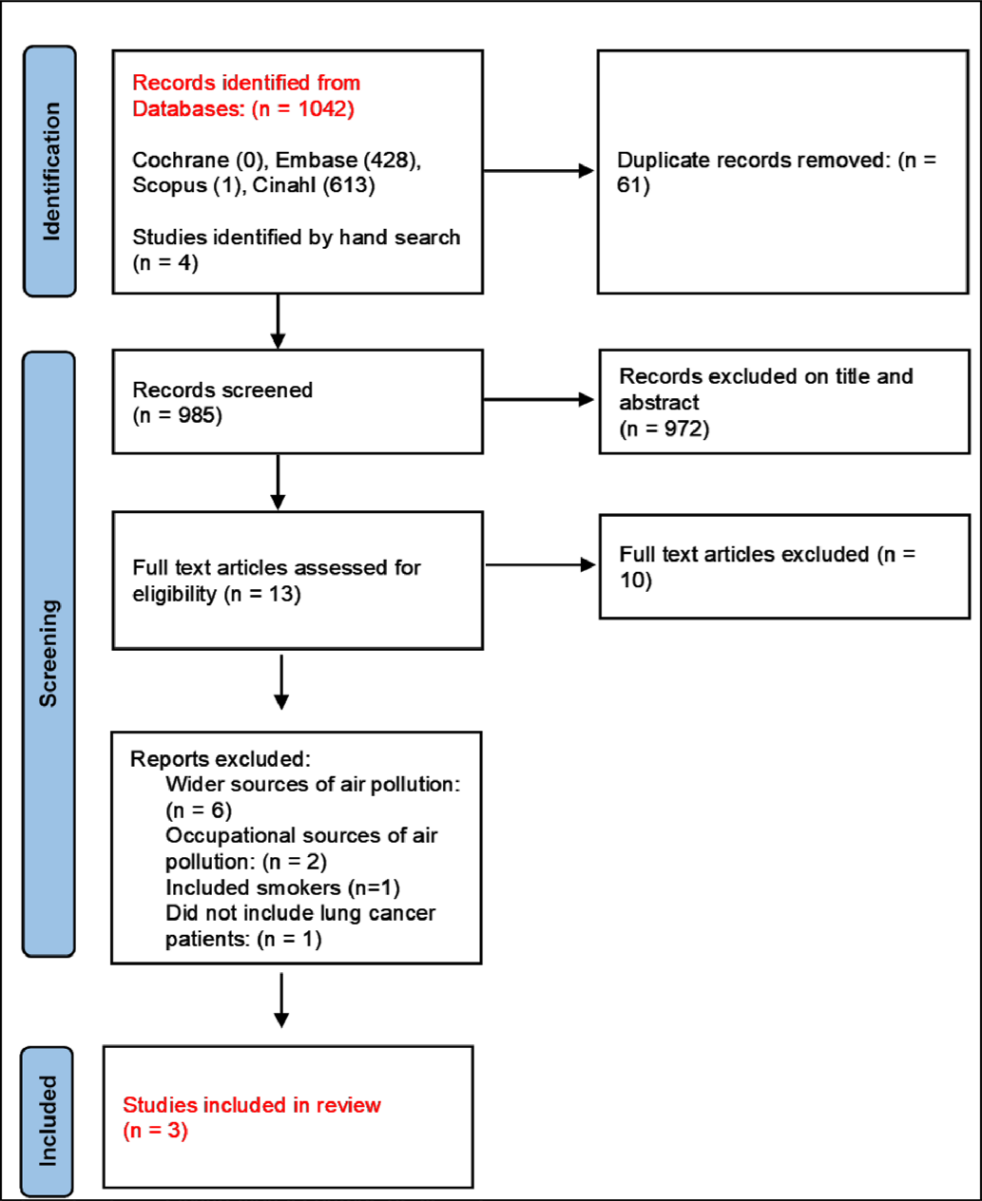
Flow chart of study selection, with reasons for exclusion.

The first two authors (BJM and RMM) independently screened all papers retrieved from the database searches by title and abstract. Screening decisions were blinded until all papers had been screened. Any differences of opinion were resolved by discussion and referring to the inclusion/exclusion criteria. The results of the search, the selection process and the number of studies are reported in figure 2.

### Data extraction

Using a bespoke extraction form, the following data from each retained study were extracted; author, publication year, study region, study design, participant characteristics, definition of exposure, confounding factors and key findings. This extraction form can be found in online supplemental appendix 2.

One author (BJM) extracted the data and a second author (RMM) double-checked data accuracy.

### Quality of evidence assessment

We assessed for quality of evidence in the selected studies using the Critical Appraisal Skills Programme (2024) (CASP) tool for case–control studies.^36^ This tool has been designed to help researchers assess whether a study is valid, has reported clear results and whether those results are applicable to the wider population. Study quality was assessed with a bespoke table designed to assist in data extraction. Examples of questions used by CASP to assess study quality are: ‘Did the study address a clearly focused issue?’, ‘Was the study population clearly defined?’, ‘Where was the study conducted?’, ‘What were the participant characteristics?’ and ‘Have the authors taken account of the potential confounding factors in the design and/or in their analysis?’. Quality assessment is available in online supplemental appendix 3.

### Data synthesis and analysis

To answer the research question, only studies that investigated long-term exposure on LCINS outcomes were included for narrative synthesis.

Retained studies had used different exposure assessment methods which were sufficiently heterogeneous to preclude meta-analysis. Each study also designed their own method of measuring cooking fume exposure, which could not be reliably pooled. Table 2 shows the methods used in each study and the test of homogeneity for meta-analysis. Narrative synthesis can be described as a textual approach to analysis of themes and relationships between studies and is appropriate where studies are too methodologically diverse to combine in a meta-analysis.^37^

**Table 2:** Meta-analysis test of homogeneity in main study findings

Study	Dose:Response	Exposure measurement	Extractor fan use
Chen *etal* ^9^	Positive dose:response shown:OR of lung cancer risk across increasing levels of cooking time-years.	Estimated cooking-time-years:Average cooking episodes per day over number of years of cooking.	Fume extractor use ratio:Total number of years of fume extractor hood use by total number of cooking years.
Yu *et al*^28^	Positive dose:response shown:OR assessed by cumulative exposure to cooking fumes and lung cancer risk, with results showing that different methods of frying were associated with different levels of risk.	Estimated cooking-dish-year:Frequency and duration of cooking episodes, using three cooking methods (stir frying, pan frying and deep frying).Total cooking dish-years were calculated by summing up years cooking by each of the three methods.	Use of extractor fan was assessed as ‘ever’ or ‘never’.
Ko *et al*^27^	Positive dose:response shown:OR calculated by number of meals cooked daily.OR of cases who waited until fumes were emitted from the oil before adding food.	Estimated number of dishes cooked:Number of meals cooked daily over number of years. Number of dishes cooked daily was found to be a greater indicator of risk than the number of cooking years.	The authors reported the effect of cooking without using a fume extractor, rather than the effect of using one.

## Results

### Study selection

Rayyan was used to facilitate processing and screening of papers. Database searching produced 1042 papers. Four additional papers were identified after searching reference lists. After duplicates were removed, 981 papers remained for screening, of which 972 papers were excluded based on the title and abstract. 13 full-text articles were assessed for eligibility and of these, 10 were excluded based on the following criteria; 6 focused on wider sources of air pollution including secondhand smoke and ambient air pollution, 2 studied occupational sources of air pollution, 1 included smoking participants, 1 did not include lung cancer cases. Key characteristics and outcomes from the three studies included at the final stage are shown in online supplemental appendix 2. A narrative synthesis of the findings of these papers was then conducted.

### Narrative synthesis of findings

Chen *et al*^9^ studied the impact of cooking oil fume (COF) exposure and fume extractor use on lung cancer in non-smoking Han Chinese women in Taiwan. 1302 non-smoking female patients with confirmed lung cancer were matched to 1302 female, cancer-free non-smokers. Controls were matched by 5-year age, sex, smoking status and education level. Exposure to COF was estimated via standardised interview-administered questionnaires, including basic demographics, the ages at which the participant started and ended cooking, habit of daily cooking times, frequency of cooking methods, types of cooking oils used, fume extractor usage, family history of lung cancer, secondhand smoke exposure, history of hormone-replacement therapy, history of oral contraceptive use, homemaker status and chef status. A non-smoker was defined as (1) a subject who has never smoked or (2) a subject who has not smoked regularly (defined as smoking at least one cigarette per week) for 6 months at any given period of her lifetime. Exposure to COF was described by ‘cooking time-years’, expressed as: Σrdr×yr, to measure exposure to cooking fumes over a participant’s lifetime, where dr=average daily times of cooking in age range r and yr=years of cooking in age range r. They describe the method as a combination of cooking frequency (cooking times per day) and duration (years). Ventilation hood use was described as fume extractor use ratio, where: (total number of years of fume extractor hood use)/(total number of cooking years).

The main findings in the study by Chen *et al*. were the dose/response association between cumulative exposure to COFs and lung cancer. The adjusted model developed for analysis included 787 cases and 1109 controls. The adjusted ORs of lung cancer risk across increasing levels of cooking time-years (11–60, 61–110, 111–160 and >160) were 1.63 (95% CI 1.20 to 2.23), 1.67 (95% CI 1.12 to 2.49), 2.14 (95% CI 1.19, to 3.90) and 3.17 (95% CI 1.34 to 7.68), respectively, relative to cooking time-years ≤10 (OR=1). Second, ventilation hood use was found to have a protective effect, with long-term use more effective than medium-term use in reducing the risk of lung cancer. Long-term use (0.67≤ratio≤1) and medium-term use (0.33≤ratio<0.67) of the fume extractor were associated with a decreased risk of lung cancer, though the impact of medium-term use was not statistically significant, with adjusted ORs of 0.49 (95% CI 0.32 to 0.76) and 0.66 (95% CI 0.42 to 1.01), respectively.

Confounding factors including age, educational level, lung cancer in a first-degree relative, secondhand smoke and the use of hormone treatments were adjusted for; however, other factors such as residential radon exposure, outdoor air pollution or indoor combustions such as mosquito coils were not considered. Nor did they collect in-depth information regarding cooking behaviour, such as the number of dishes per meal, cooking methods or other factors. There was also a risk of recall bias due to the use of questionnaires rather than household air sampling.

Yu *et al*^28^ explored the dose/response relationship between cooking fume exposures and lung cancer among Chinese women in Hong Kong. They studied 200 female non-smokers with confirmed lung cancer and 285 female non-smoking controls matched by 10-year age group and residential district. In-person interviews using a standardised structured questionnaire were used to collect information on regular cooking habits at each residence since childhood. Exposure to cooking fumes was described as ‘cooking dish-years’ as a measure of exposure. To take into account not only the frequency and duration of cooking, but also three different cooking methods (stir frying, pan frying and deep frying) they created: $\sum_{i=1}^{k}d_{i}Y_{i}$, where k=number of residences lived, di=average number of dishes cooked daily by the selected method at the ith residence, and yi=years of cooking at the ith residence. A participant’s total cooking dish-years was calculated by summing up all her years cooking by each of the three methods, giving researchers a reliable measure of her lifetime cooking fume exposure. Use of extractor fan was assessed as ‘ever’ or ‘never’ within the administered questionnaire.

Yu *et al* found a positive dose/response relationship between cumulative exposure to cooking fumes and lung cancer risk, with results showing that different methods of frying were associated with different levels of risk. The risk of LCINS increased with levels of cooking dish-years as described above, using model 1 which adjusted for five confounders including age and education, where cooking dish-years ≤50=OR 1. While the dose/response relationship does not achieve statistical significance until exposure passes 100 cooking dish-years, this trend continues with increased exposure; (51–100=1.31, 95% CI 0.81 to 2.11, 101–150=OR 2.80, 95% CI 1.52 to 5.18, 151–200=OR 3.09, 95% CI 1.41 to 6.79 and >200=OR 8.09, 95% CI 2.57 to 25.45).

Deep-frying was associated with the highest risk (per 10 dish year) with an OR of 2.56 (95% CI 1.31 to 5.00). This was followed by pan-frying (OR 1.47; 95% CI 1.27 to 1.69) and stir-frying (OR 1.12; 95% CI 1.07 to 1.18). The use of ventilation hoods was not found to have a significant impact on the risk of developing lung cancer comparing those who had ever used an extractor fan with those who had not (OR 0.94; 95% CI 0.43 to 2.02). While the comparator was not explicitly stated in the paper, it can be assumed to be those who do not fry or heat their pan to high temperatures.

Confounding factors including environmental tobacco smoke, residential radon, other sources of indoor air pollutants (eg, mosquito coils or incense burning), family history of lung disease and sociodemographic factors were adjusted for. Some potential influences such as interviewer bias, outcome misclassification (eg, control subjects with undiagnosed lung cancer) were acknowledged.

Ko *et al*^27^ explored the link between Chinese food cooking and lung cancer in Taiwanese female non-smokers, studying 131 non-smoking females with a confirmed lung cancer diagnosis, 252 hospital controls with admissions unrelated to smoking and 262 community controls. Controls were matched by age (within 2 years), smoking status and geographical area. Each case had one hospital and two community controls. Trained interviewers collected data on factors including demographic and socioeconomic characteristics, smoking history, general air pollution within the home, ventilation conditions and cooking habits. A non-smoker was defined as someone who had not smoked one cigarette in her lifetime. Exposure to cooking fumes was measured in the number of meals cooked daily, and cooking years began at the age that a woman started cooking daily.

Using this measure, they found that the number of dishes cooked daily was a greater indicator of risk than the number of cooking years, citing a threefold increased risk of lung cancer among women who cooked three meals per day compared with those who cooked one (OR 3.1 (95% CI 1.6 to 6.2)). Another finding was that cases who waited until fumes were emitted from the oil before adding food had a significantly higher risk of lung cancer when compared with hospital controls (OR 2.5, 95% CI 1.4 to 4.3). The authors reported the effect of cooking without using a fume extractor, finding that not using an extractor led to a significantly higher risk of lung cancer, with OR 5.4 (95% CI 2.7 to 10.8) when compared with hospital controls, and OR 2.2 (95% CI 1.3 to 3.8) when compared with community controls.

While confounding factors such as occupation, passive smoking and residential area were included in the questionnaire, these were not discussed within the paper.

## Discussion

### Summary of findings

There is increasing recognition of individuals who have never smoked developing lung cancer, especially in women and non-white ethnic groups.^38^ This narrative review found a significant knowledge gap regarding the role of cooking-related indoor air pollution on the risk of LCINS in HICs. The current evidence, based on only three studies of ethnically Chinese women, suggests an association between exposure to cooking fumes and the development of LCINS. In addition, the use of extractor fans is likely associated with a reduced risk of developing LCINS. These findings, however, need to be replicated in much wider demographic and geographic settings before a possible conclusion can be made.

The use of fume extractor hoods to reduce exposure to cooking fumes was discussed in all three papers, though there was sufficient heterogeneity within the reporting methods to preclude meta-analysis. Chen *et al* suggested that fume extractor hoods could reduce the risk of lung cancer by 50%–60% if used regularly,^9^ though Ko *et al* cautioned that the effectiveness of hoods could be dramatically reduced if not installed or used correctly.^27^ A meta-analysis of the use of fume extractor hoods in China found a statistically significant protective effect associated with their use (OR 0.54, 95% CI 0.48 to 0.61).^39^

### Limitations

One clear limitation of the retrieved studies was that all three relied on questionnaires and the recollection of participants, rather than actively monitoring concentrations of household air pollutants while cooking. None of the papers included the questionnaires used in their publications. While questionnaires can be very useful tools to capture exposures, they are subject to recall bias, especially when studying mundane household chores, such as daily cooking, over an entire lifespan.^40 41^

None of the studies accounted for the effects of outdoor air pollution, which is known to have a significant impact on health, is a confirmed risk factor for lung cancer, and can affect the quality of household air.^3 5 42^ Air quality is a major global concern^42^ and has been linked with respiratory-related hospitalisation episodes in both Hong Kong^43 44^ and Taiwan.^45 46^ Given that the studies used questionnaires to assess exposure over a lifetime, it may have been impossible to adjust for the specific impact of historical outdoor air quality, though this is something that future studies should consider addressing as a confounding factor.

Asia has been found to have among the highest incidence of LCINS in female patients, with rates reaching their peak in the area of Xuanwei in China.^11^ This review was restricted to HICs as described by The World Bank, and even so, all eligible papers investigated Chinese women in Taiwan and Hong Kong, who were using traditional cooking methods. This raises questions around applicability to other ethnic groups and other HICs. The lack of papers retrieved also highlights the paucity of studies into the impact of household air pollution on LCINS in HICs.

While no language limitations were placed on our searches, it is likely that studies published in non-English journals were not returned as they do not provide key words or abstract in English. Therefore, it is possible that some relevant studies have not been included in this review.

### Implications for practice and research

Despite the fact that household air pollution affects every household worldwide, to date the majority of research into this area has been conducted in LMICs, focusing on issues such as the use of solid fuels for cooking and heating, or the use of polluting cookstoves and open fires within the home.^8 10 16^ While these studies produce valuable information regarding the risks faced by the participants involved, and hence have promoted global health overall, they are not widely applicable to HICs where gas or electric cooking and heating systems are more prevalent.

There is a growing interest in the issue of household air quality in Europe and North America in recent years, with public interest in the health impact of issues such as mould and wood-burning stoves.^2 3 47^ As such, we can expect greater investigation into how household air affects the risk of LCINS in future studies.

Additional research is particularly needed to help understand how cooking-related household air pollution impacts the risk of developing LCINS for women of different ethnicities in HICs, which could inform policies in addressing health inequality issues.

## Conclusions

Exposure to cooking fumes is linked to lung cancer due to the levels of carcinogens that can be produced during the heating of oils to high temperatures.^8 16 17^ VOCs which are generated by cooking oils have been shown to be mutagenic, containing PACs, aldehydes, carbonyl compounds and other mutagens.^8 17 48^

This review of three studies found a possible association between exposure to cooking fumes and the risk of developing LCINS in high-income settings. This corroborates the substantial body of evidence that links cooking fume exposure to LCINS in LMICs, with definitive confirmation of the exposure hazards.

## Supplementary material

10.1136/bmjopen-2024-093870 online supplemental file 1

10.1136/bmjopen-2024-093870 online supplemental file 2

10.1136/bmjopen-2024-093870 online supplemental file 3

## Data Availability

All data relevant to the study are included in the article or uploaded as supplementary information.
