# Supplementary material for: Relationship between household air pollution and lung cancer in never smokers in high-income countries: a systematic review
Source: BMJ Open. 2025 Jun 20;15(6):e093870. doi: 10.1136/bmjopen-2024-093870 (PMC12182138; doi:10.1136/bmjopen-2024-093870)
Supplement: online supplemental file 1 [file bmjopen-15-6-s001.docx]

Appendix 1: Search strings used for Embase, Scopus, Cochrane Library and CINAHL

Embase

| Subject | MeSH terms |
| --- | --- |
| Smoking status | ((no* adj2 smoke*) or (never adj2 smoke*)).mp. |
| Lung cancer | (Lung cancer in never smokers or LCINS or NSCLC).mp.  (lung adj2 (ca* or neo* or adeno* or malig* or tumo?r)).mp.  (pulmonary adj2 (ca* or neo* or adeno* or malig* or tumo?r)).mp. |
| Indoor air pollution | (Air Pollut* or Indoor or emission* or exposur*).mp.  ((house* or indoor*) adj2 air adj2 (pollut* or quality)).mp.  ((indoor* or house* or domestic* or home) adj3 (fire* or smok* or woodsmok*)).mp.  ((combust* or burn*) adj3 (stove* or wood or kerosene or biomass or coal or charcoal or dung or crop waste* or agricultur* residu*)).mp.  (stove* or cookstove* or kitchen ventilat* or combustion or house construction or solar).mp.  (vent* adj5 (home* or living space* or household* or kitchen* or cook* or stove* or window* or hood* or grate*)).mp.  (particulate matter or PM or ultrafine partic* or UFP).mp.  (acenaphthene or acenaphthylene or anthracene or benzanthracene or benzene or C6H6 or benzopyrene or benzofluoranthene or benzoperylene or benzofluoranthene or butadiene or carbon monoxide or chrysene or fluoranthene or C16H10 or fluorene or formaldehyde or naphthalene or nitric oxide or nitrogen dioxide or NO2 or nitrogen oxide* or NOx or nitrous acid or phenanthrene or polycyclic aromatic hydrocarbon* or PAH).mp. |
| Study type | (Case-Control Studies or Cross-Over Studies or Cohort Studies or Longitudinal Studies).mp. |

Scopus

| Subject | MeSH terms |
| --- | --- |
| Smoking status | no* AND smoke OR never AND smoke* |
| Lung cancer | lung AND cancer AND in AND never AND smokers OR lcins OR nsclc AND lung AND ca* OR neo* OR adeno* OR malig* OR tumour OR tumor OR pulmonary AND ca* OR neo* OR adeno* OR malig* OR tumour OR tumor |
| Indoor air pollution | Indoor* air AND pollut* |
| Study type | Case-Control Studies OR Cross-Over Studies OR Cohort Studies OR Longitudinal Studies |

Cochrane Library

| Subject | MeSH terms |
| --- | --- |
| Smoking status | "non-smoker" or "non smoking" |
| Lung cancer | "Lung cancer in never smokers" or "LCINS" or "NSCLC" |
| Indoor air pollution | "indoor air quality" or "indoor air pollution" or "particulate matter" or "pm" or "ventilation" |
| Study type | Case-Control Studies OR Cross-Over Studies OR Cohort Studies OR Longitudinal Studies |

CINAHL

| Subject | MeSH terms |
| --- | --- |
| Smoking status | non-smoker or never smoker |
| Lung cancer | (lung AND cancer AND in AND never AND smokers) OR (lcins OR nsclc) or (lung AND ca* OR neo* OR adeno* OR malig* OR tumour OR tumor) OR (pulmonary AND ca* OR neo* OR adeno* OR malig* OR tumour OR tumor) |
| Indoor air pollution | (indoor air quality) or (indoor air pollution) or (particulate matter) or pm or ventilation or smok* or coal or biomass or burn or (ultrafine particul*) or UFP or (polycyclic aromatic hydrocarbon* or PAH) |
| Study type | Case-Control Studies OR Cross-Over Studies OR Cohort Studies OR Longitudinal Studies |

Search Results

| Database | Papers returned |
| --- | --- |
| Cochrane Library | 0 |
| Embase | 428 |
| Scopus | 1 |
| Cinahl | 613 |
