# Supplementary material for: Relationship between household air pollution and lung cancer in never smokers in high-income countries: a systematic review
Source: BMJ Open. 2025 Jun 20;15(6):e093870. doi: 10.1136/bmjopen-2024-093870 (PMC12182138; doi:10.1136/bmjopen-2024-093870)
Supplement: online supplemental file 2 [file bmjopen-15-6-s002.docx]

Appendix 2: Extraction form

| **Title** | **Author (Year)** | **Country** | **Study Design** | **Participant Characteristics** | **Definition of Exposure** | **Confounding factors** | **Key Findings** |
| --- | --- | --- | --- | --- | --- | --- | --- |
| **Impact of cooking oil fume exposure and fume extractor use**  **on lung cancer risk in non-smoking**  **Han Chinese women** | Chen et al (2020) | Taiwan | Case-control.  Interview-administered questionnaires. | Confirmed lung cancer patients, who were non-smoking Chinese females  >18 yrs (n= 1,302)  Controls, cancer-free non-smoking Chinese females >18 yrs (n= 1,302) | Exposure to COF was described by “cooking time-years”, expressed as: Σrdr × yr, to measure exposure to cooking fumes over a participant’s lifetime | Adjusted for confounding factors including age, educational level, lung cancer in a first-degree relative, second hand smoke and the use of hormone treatments.  Acknowledged confounders not measured, such as the number of dishes per meal, cooking methods or other factors. | Increased risk of LCINS with higher cooking time years, with clear dose:response trend: aOR 1.63 (95% CI 1.20, 2.23) at lowest, 3.17 (95% CI 1.34, 7.68) at highest.  Extractor hoods reduce the risk of LCINS in medium-term and high-term use, with aOR of 0.66 (95% CI 0.42, 1.01) and 0.49 (95% CI 0.32, 0.76) respectively. |
| **Dose-Response relationship between cooking fume exposures and lung cancer among Chinese non-smoking women** | Yu et al (2006) | Hong Kong | Case-control.  Interview-administered questionnaires. | Confirmed lung cancer patients, who were non-smoking Chinese females  30-79 yrs (n= 200)  Controls, cancer-free non-smoking Chinese females, age matched (n= 285) | Exposure to cooking fumes was described as “cooking dish-years” as a measure of exposure to cooking fumes.  σ𝑑𝑖𝑌𝑖𝑘𝑖=1, where k = number of residences lived, d*i*= average number of dishes cooked daily by the selected method at the *i*th residence, and y*i*= years of cooking at the *i*th residence. | Confounding factors including environmental tobacco smoke, residential radon, other sources of indoor air pollutants, family history of lung disease and sociodemographic factors were adjusted for. | Cooking-dish-years  Dose:response trend  Risk of LCINS varies with style of frying:  Deep fry: OR 2.56 (95% CI 1.31, 5)  Fry: 1.47 (95% CI 1.27, 1.69)  Stir fry: OR 1.12 (95% CI 1.07, 1.18) |
| **Chinese food cooking and lung cancer in women non-smokers** | Ko et.al. (2000) | Taiwan | Case-control.  Interview-administered questionnaires. | Confirmed lung cancer patients, who were non-smoking Taiwanese females (n = 131)  Hospital controls; cancer-free, non-smoking Taiwanese females (n = 252)  Community controls; cancer-free, non-smoking Taiwanese females (n = 262) | Exposure to cooking fumes was measured in the number of meals cooked daily. | While confounding factors such as occupation, passive smoking and residential area were included in the questionnaire, these were not discussed within the paper. | Dose-response trend: 3 meals per day OR 2.8 (95% CI 1.2, 6.3)  Risk is higher when oil is heated until smoking before cooking.  Highest risk group were those who waited until oil was smoking and did not use extractor hood (OR 3.2 –12.2). |
