# Supplementary material for: Relationship between household air pollution and lung cancer in never smokers in high-income countries: a systematic review
Source: BMJ Open. 2025 Jun 20;15(6):e093870. doi: 10.1136/bmjopen-2024-093870 (PMC12182138; doi:10.1136/bmjopen-2024-093870)
Supplement: online supplemental file 3 [file bmjopen-15-6-s003.docx]

Appendix 3: CASP Quality of Evidence template

| Authors, (year) | Did the study address a clearly focused issue? | Did the authors use an appropriate method to answer their question? | Were the cases recruited in an acceptable way? | Were the controls selected in an acceptable way? | Was the exposure accurately measured to minimise bias? | Aside from the experimental intervention, were the groups treated equally? | Have the authors taken account of the potential confounding factors in the design and/or in their analysis? | How large was the treatment effect? | Do you believe the results? | Can the results be applied to the local population? | Do the results of this study fit with other available evidence? |
| --- | --- | --- | --- | --- | --- | --- | --- | --- | --- | --- | --- |
| Chen (2020) | Y | Y | Y | Y | Y | Y | Y | N/A | Y | Y | Y |
| Yu et al (2006) | Y | Y | Y | Y | Y | Y | Y | N/A | Y | Y | Y |
| Ko et.al. (2000) | Y | Y | Y | Y | Y | Y | Unsure | N/A | Y | Y | Y |
