# Supplementary material for: Relationship between household air pollution and lung cancer in never smokers in high-income countries: a systematic review
Source: BMJ Open. 2025 Jun 20;15(6):e093870. doi: 10.1136/bmjopen-2024-093870 (PMC12182138; doi:10.1136/bmjopen-2024-093870)
Supplement: Supplementary file 4 [file bmjopen-2024-093870.reviewer_comments.pdf]

PEER REVIEW HISTORY

BMJ Open publishes all reviews undertaken for accepted manuscripts. Reviewers are asked to complete a checklist review form and are provided with free text boxes to elaborate on their assessment. These free text comments are reproduced below.

ARTICLE DETAILS

Title (Provisional)

The relationship between household air pollution and lung cancer in never smokers in high income countries: A systematic review

Authors

McAllister, Bría Joyce; Malhotra Mukhtyar, Rushad; Cai, Samuel; Brown, Karen; Lock, Selina; Khan, Sam

---

VERSION 1 - REVIEW

Reviewer 1

Name Taylor, Rachel

Affiliation University College London Hospitals NHS Foundation Trust, CNMAR

Date 27-Jan-2025

COI As noted to the managing editor, I am the PhD students clinical supervisor, i.e., responsible for supporting her career development as a nurse. I did not have any input into this review and other than noting it was being done, it did not form any part of our supervision discussion. As requested by the managing editor, I note this confidentially as part of my review.

Thank you for asking me to review this systematic review reporting the influence of household pollutants on the risk on lung cancer in never-smokers. I offer the following suggestions to strengthen the reporting of this paper.

1. Strengths and weaknesses – these are key aspects of a review you would expect. I would suggest reading the guidance and putting more reflection onto what are novel to this review.

2. Introduction – abbreviations need to be in full the first time they are used (LCINS, UK). Figure is not referred to in the text and should be linked o some explanations. It this has been taken from another source, this needs to be referenced.

3. The PECO table is not referred to in the text and has a PECO title then a table title underneath. I would suggest putting table titles at the top of the tables and figure titles underneath. Also ensuring they are all included in the text.

4. The searches should be included as an appendix. I note they are at the end of the paper but the fact they are a supplemental file or appendix needs to be added to the text in the paper and these need a heading so it is clear these are supplemental.

5. Figure 2 is not referred to in the text.

6. Table 2 is referred to as the extraction form when it is more than that, and should be referred to again in the relevant results section.

7. Sentences should not start with a number, it needs to be in words in full.

8. As the review used papers with similar study designs, was it possible to undertake any form of collective analysis (such as metanalysis) using the available data; or was it possible to present the results in alongside each other so a comparison could be made – it is currently very text heavy.

9. The discussion has a change in referencing style and needs to conform to the journal style. The summary needs to provide more detail on where there is convergence/congruence in results of the three cited studies. There is a lot of other literature provided that is summarising what else is know and it is unclear why these papers were not included. The authors should follow the journal style guide on the length of the discussion and only include literature that links to the finding of the review.

10. The limitations discusses the limitations of the included literature, which I would suggest is more of the discussion. The limitations should be the limitations of the review (I would suggest that this is all publications written in English and as all the papers are from the Western Pacific Region, there are potentially going to be papers that are missing as they are published in non-English journals that don't provide English abstracts).

Reviewer **2**

Name **Balmes, JR**

Affiliation **University of California, Berkeley, Division of Occupational and Environmental Medicine**

Date **19-Feb-2025**

COI **None**

GENERAL COMMENTS

This manuscript is a well-written report of a systematic review of indoor exposure to cooking emissions and lung cancer in nonsmoking women (LCINS) in high-income countries. The manuscript has multiple strengths including PROSPERO protocol registration, careful review

of the literature following PRISMA guidelines, double-author screening, double-checked data accuracy, appropriate selection of studies to be reviewed, and study quality assessment with CASP. Despite these strengths there are several important limitations acknowledged by the authors. Only three studies met criteria for review, all three involved only Chinese women using traditional cooking methods (two from Taiwan and one from Hong Kong), and the total number of participants was relatively small (~3700). Thus, the generalizability of the results of the review is problematic. The secondary finding that use of cooking ventilation may reduce risk of LCINS is potentially impactful. The authors call for additional research on the risk of LCINS and cooking emissions is an important one.

SPECIFIC COMMENTS

p. 4, line 14 Should be "infiltration" here rather than filtration to avoid confusion.

p. 4, last paragraph The authors should make clear that the content of this paragraph applies almost completely to household air pollution due to the combustion of solid fuels for cooking and heating in LMICs.

p. 10, line 32 and later Should be "ventilation" hood rather than ventilator to avoid confusion with devices used for mechanical ventilation of patients.

p. 15, line 26 Should be "historical" at the than historic.

VERSION 1 - AUTHOR RESPONSE

Reviewer: 1
Prof. Rachel Taylor, University College London Hospitals NHS Foundation Trust, University College London
Comments to the Author:
Thank you for asking me to review this systematic review reporting the influence of household pollutants on the risk on lung cancer in never-smokers. I offer the following suggestions to strengthen the reporting of this paper.
1. Strengths and weaknesses – these are key aspects of a review you would expect. I would suggest reading the guidance and putting more reflection onto what are novel to this review.

The strengths and weaknesses have been reviewed and edited to contain 5 bullet points, including one limitation.

2. Introduction – abbreviations need to be in full the first time they are used (LCINS, UK). Figure is not referred to in the text and should be linked o some explanations. It this has been taken from another source, this needs to be referenced.

Abbreviations have been written in full. A line has been added to the text to link to figure 1. This figure was created for this paper and has not been taken from another source.

3. The PECO table is not referred to in the text and has a PECO title then a table title underneath. I would suggest putting table titles at the top of the tables and figure titles underneath. Also ensuring they are all included in the text.

The PECO statement and explanation has been referenced in the text, and the title has been adjusted accordingly.

4. The searches should be included as an appendix. I note they are at the end of the paper but the fact they are a supplemental file or appendix needs to be added to the text in the paper and these need a heading so it is clear these are supplemental.

The search strings have been referenced in the text, directing the reader towards supplemental material in Appendix 1.

5. Figure 2 is not referred to in the text.

This has now been referred to in the text.

6. Table 2 is referred to as the extraction form when it is more than that, and should be referred to again in the relevant results section.

This has been addressed in the text, directing the reader towards supplemental material in Appendix 2.

7. Sentences should not start with a number; it needs to be in words in full.

This has been addressed in the text.

8. As the review used papers with similar study designs, was it possible to undertake any form of collective analysis (such as metanalysis) using the available data; or was it possible to present the results in alongside each other so a comparison could be made – it is currently very text heavy.

Unfortunately, as all three studies used different techniques for data collection, analysis and reporting, it was not possible to perform a meta-analysis on any of the results. This was attempted during the analysis phase, but on advice from a statistician it was felt that a narrative review was the most appropriate way to present the results.

A table has been added to show the methodology used in studies, and the test of homogeneity for meta-analysis.

9. The discussion has a change in referencing style and needs to conform to the journal style. The summary needs to provide more detail on where there is convergence/congruence in results of the three cited studies. There is a lot of other literature provided that is summarising what else is know and it is unclear why these papers were not included. The authors should follow the journal style guide on the length of the discussion and only include literature that links to the finding of the review.

Significant changes have been made to the discussion section to bring it in line with the journal style guide. Literature on the wider topic has been removed as it is not relevant to this specific review. The discussion has been shortened and edited to make it clearer, more relevant and easier to read.

10. The limitations discuss the limitations of the included literature, which I would suggest is more of the discussion. The limitations should be the limitations of the review (I would suggest that this is all publications written in English and as all the papers are from the Western Pacific Region,

there are potentially going to be papers that are missing as they are published in non-English journals that don't provide English abstracts).

The section discussing the limitations of the included literature has been moved into the discussion section where it is more appropriately placed. The Limitations section has been rewritten.

Reviewer: 2
Prof. JR Balmes, University of California, Berkeley
Comments to the Author:
GENERAL COMMENTS
This manuscript is a well-written report of a systematic review of indoor exposure to cooking emissions and lung cancer in nonsmoking women (LCINS) in high-income countries. The manuscript has multiple strengths including PROSPERO protocol registration, careful review of the literature following PRISMA guidelines, double-author screening, double-checked data accuracy, appropriate selection of studies to be reviewed, and study quality assessment with CASP. Despite these strengths there are several important limitations acknowledged by the authors. Only three studies met criteria for review, all three involved only Chinese women using traditional cooking methods (two from Taiwan and one from Hong Kong), and the total number of participants was relatively small (~3700). Thus, the generalizability of the results of the review is problematic. The secondary finding that use of cooking ventilation may reduce risk of LCINS is potentially impactful. The authors call for additional research on the risk of LCINS and cooking emissions is an important one.

SPECIFIC COMMENTS
p. 4, line 14 Should be "infiltration" here rather than filtration to avoid confusion.

This has been changed.

p. 4, last paragraph The authors should make clear that the content of this paragraph applies almost completely to household air pollution due to the combustion of solid fuels for cooking and heating in LMICs.

This paragraph has been edited to make clear that the majority of studies focus on LMICs and may not be directly applicable to HICs.

p. 10, line 32 and later Should be "ventilation" hood rather than ventilator to avoid confusion with devices used for mechanical ventilation of patients.

This has been changed.

p. 15, line 26 Should be "historical" at the than historic.

This has been changed.

Reviewer: 1
If you have selected 'Yes' above, please provide details of any competing interests.: As noted to the managing editor, I am the PhD students clinical supervisor, i.e., responsible for supporting her career development as a nurse. I did not have any input into this review and other than noting it was being done, it did not form any part of our supervision discussion. As requested by the managing editor, I note this confidentially as part of my review.

Reviewer: 2
If you have selected 'Yes' above, please provide details of any competing interests.: Not applicable

VERSION 2 - REVIEW

Reviewer 1

Name Taylor, Rachel

Affiliation University College London Hospitals NHS Foundation Trust, CNMAR

Date 14-Apr-2025

COI

Thank you for addressing my concerns. These have mostly been addressed and just have a few minor suggestions to improve the reporting of this paper. The new table (table 2), I'm not sure the final row is required - this could be simply stated in the text. The references in the discussion need to be updated to the journal format. The placement of the limitations subheading needs to align to where the limitations start

VERSION 2 - AUTHOR RESPONSE

Reviewer: 1
Prof. Rachel Taylor, University College London Hospitals NHS Foundation Trust, University College London
Comments to the Author:
Thank you for addressing my concerns. These have mostly been addressed and just have a few minor suggestions to improve the reporting of this paper. The new table (table 2), I'm not sure the final row is required - this could be simply stated in the text.

The table has been edited to remove the final row. The rationale for performing narrative review rather than meta-analysis has been stated in the text.

The references in the discussion need to be updated to the journal format.

The references have been updated to the journal format.

The placement of the limitations subheading needs to align to where the limitations start

This has been adjusted accordingly
